# A Manual of Practical Medicine

**Published:** 1858-11

**Authors:** 


ART. VII.—*A Manual of Practical Medicine.* By T. H. TANNER, M.D., Author of a Manual of Clinical Medicine and Physical Diagnosis, etc. etc. First American from the third revised and improved London edition. 12mo. pp. 398. Lindsay and Blakiston: Philadelphia, 1858.

GREATLY in contrast with the treatise of Dr. Wood is the unpretending manual of Dr. Tanner; and yet we must confess a great liking for all the little works of this author; for, although they are not intended for those more advanced in medical literature, yet they are of great utility to the student and to the practitioner who has not time to read, or will not read, the more elaborate treatises on practical medicine. We do not like the author's classification of the varieties of cancer, nor his description of keloid; but on the whole, without any waste of words or of space, his delineations of the pathology and treatment of disease are good, and his style is easy, straight-forward, and to the point. We should have wished the compass of the work to be somewhat more extended; for it is rather too much of a compendium, and here and there we find that whilst too much space is devoted to one disease, too little is given to another. The manuals of Dr. Tanner are rather better than we generally find them; and, although they lay no claim to originality, it is easy to see that the author is thoroughly conversant with the subjects he discusses. The appendix of formulæ alone occupies sixty-two pages; too much room when contrasted with the rest of the work. It would, in our opinion, have been better to have omitted them altogether, and to have filled up the space with more extended descriptions of diseases.

